# MeVO: To Treat or Not To Treat? Review of the Latest Evidence

**DOI:** 10.1007/s11910-026-01515-0

**Published:** 2026-09-18

**Authors:** Ossama Yassin Mansour, Ahmed Ossama, Nada Nasr, Eman Hamdy, Faisal Alghamdi, Nadia Hammami

**Affiliations:** 1 Department of Neurology, Stroke and Neurointervention Center, Faculty of Medicine, Alexandria University, 23121 Alexandria, Egypt; 2 Interventional Neuroradiology Department, King Abdullah Medical City, Makkah, Saudi Arabia; 3 Diagnostic and Interventional Neuroradiology, National Institute of Neurology Tunis, Tunis, Tunisia

**Keywords:** Medium vessel occlusion, Distal medium vessel occlusion, Endovascular thrombectomy, Patient selection, Cost-effectiveness, Global stroke care

## Abstract

**Purpose of Review:**

Endovascular thrombectomy (EVT) is standard care for large vessel occlusion, but its role in medium vessel occlusion (MeVO) is contested. We appraise randomized and real-world evidence and ask whether, and in whom, MeVO should be treated.

**Recent Findings:**

DISTAL, ESCAPE-MeVO and DISCOUNT found no functional benefit from adding EVT to medical treatment, with more hemorrhage and serious adverse events; DISTAL’s 12-month results were also neutral. ORIENTAL-MeVO, restricted to deficits of NIHSS ≥ 6 within 24 h, reported greater functional independence with EVT (58.6% versus 46.6%). The MEMENTO registry (164 patients, 15 Middle East and North Africa centres) reproduced trial-level outcomes — 69% recanalization, 54% independence, 3% symptomatic hemorrhage — while modelled cost-effectiveness ratios of $26,875–71,333/QALY exceeded regional thresholds. DISTALS achieved high reperfusion without symptomatic hemorrhage using a MeVO-dedicated stentretriever.

**Summary:**

Routine MeVO thrombectomy is unjustified. Benefit concentrates in moderate-to-severe deficits, eloquent territory, salvageable tissue and technically accessible primary occlusions.

## Introduction

For a decade the reperfusion story was one of expansion: proximal occlusions first, then late windows, large cores, basilar occlusions and pre-existing disability. Medium vessel occlusion (MeVO) looked like the obvious next frontier. MeVO accounts for 25–40% of acute ischemic strokes and carries substantial residual morbidity under best medical management [1, 2], a 2020 consensus statement framed distal, medium vessel occlusions as a promising device target [3, 4], and a subgroup analysis of the HERMES collaboration suggested benefit in M2 occlusions [5]. Medical therapy is demonstrably imperfect: intravenous thrombolysis (IVT) is constrained by eligibility criteria and recanalizes only about half of MeVOs [6].

The counterargument was never weak. Distal arteries are smaller, more tortuous, more mobile and thinner-walled than the M1 segment; the territory at risk is smaller; deficits are milder on average; and the ceiling for improvement is therefore lower. Until recently neither European nor American guidelines gave a clear recommendation, and practice ranged from treating nearly every MeVO to almost never intervening [7].

Two further gaps shaped the debate. First, evidence came overwhelmingly from high-volume centres in North America and Europe, leaving real-world effectiveness in resource-variable health systems — where stroke burden is high and infrastructure heterogeneous — largely unmeasured [2]. Second, no randomized trial has reported an economic endpoint, even though MeVO intervention competes directly for the resources that fund stroke units, large vessel occlusion (LVO) capacity and prevention.

Between February 2025 and July 2026 five randomized trials arrived with, at first glance, contradictory answers [8–12]. In parallel, our group reported the MEMENTO registry, a 15-centre analysis of consecutive distal medium vessel occlusion (DMVO) thrombectomies across the Middle East and North Africa (MENA) region, with a prespecified economic analysis [2]. We declare that involvement at the outset: several observations below are drawn from our own data and carry the limitations of an uncontrolled registry.

This review synthesizes the randomized, real-world and economic evidence; dissects why the early trials were neutral and the latest one was not; and proposes a pragmatic framework for the question every stroke team now faces at 3 a.m.

### Defining the Target: Which Vessel, and By Which Mechanism?

Terminology remains a nuisance. “MeVO,” “DMVO” and “distal occlusion” are used interchangeably for lesions sharing little except being *not* an internal carotid, M1 or basilar occlusion; most studies define them as M2/M3, A1–A3 or P1–P3 occlusions [13]. The trials differed materially: ESCAPE-MeVO enrolled non-dominant/co-dominant M2, M3, A2, A3, P2 and P3 occlusions within 12 h, whereas DISTAL also included M4, A1 and P1 up to 24 h [7]; DISCOUNT required occlusion distal to the mid-height of the insula plus NIHSS ≥ 5 or disabling aphasia [10]. A proximal dominant M2 occlusion causing hemiplegia and aphasia has almost nothing in common — anatomically, hemodynamically or prognostically — with an isolated P3 occlusion causing a quadrantanopia. Territorial eloquence, not caliber alone, should drive reperfusion decisions [1].

A second axis of heterogeneity has been almost entirely neglected. In MEMENTO we classified DMVOs angiographically as primary (medium vessel occlusion without proximal thrombus), baseline secondary (from spontaneous fragmentation or migration of a proximal clot) and iatrogenic secondary (arising during proximal thrombectomy). Among 164 patients these accounted for 25%, 50% and 25% respectively; successful recanalization differed significantly across subtypes (78% primary, 59% baseline secondary, 68% iatrogenic; *p* = 0.046), and baseline secondary DMVO was an independent predictor of poor 90-day outcome (adjusted OR 2.08, 95% CI 1.02–4.23) [2].

This matters more than it first appears. The randomized trials enrolled, by design, patients presenting with a primary medium vessel occlusion. Yet in real-world neurointervention roughly half of medium vessel thrombectomies are performed on secondary occlusions — including the operator standing at the table having just embolized an M3 branch during an otherwise successful M1 case. Applying a neutral trial result to that intraprocedural decision is a category error: the population, the timing, the tissue at risk and the counterfactual are all different. Table 1 summarizes the randomized trials alongside the registry comparator.

**Table 1:** Randomized trials of thrombectomy for medium/distal vessel occlusion stroke, with real-world registry comparator

Study (year)	Design; *n*	Occlusion sites; window	Severity; median age	Primary outcome	Result	Key safety
ESCAPE-MeVO (2025)	RCT, 5 countries; 530	Non-dominant/co-dominant M2, M3, A2, A3, P2, P3; ≤12 h	NIHSS unrestricted; 75 y	mRS 0–1 at 90 d	41.6% vs. 43.1% (aRR 0.95)	Higher sICH and mortality with EVT
DISTAL (2025; 12-month 2026)	RCT, 55 hospitals; 543	M2–M4, A1–A3, P1–P3; ≤6 h, or 6–24 h with salvageable tissue	Median NIHSS 6; 77 y	90-d mRS shift	Neutral at 90 d and 12 m (acOR 0.81, 0.59–1.12)	No excess sICH/death; no QoL gain
DISCOUNT (2025 interim; 2026 final)	RCT, 22 centres; 163 of 488 planned	MCA distal to mid-insula, A1–A3, P1–P3; ≤6 h, or ≤ 24 h with imaging	NIHSS ≥ 5 or disabling aphasia; 74 y	mRS 0–2 at 90 d	60% vs. 77% (OR 0.42, 0.2–0.88); stopped early	Any ICH 44% vs. 29%; sICH 12% vs. 6%
ORIENTAL-MeVO (2026)	RCT, 48 centres; 563	MeVO; ≤24 h (salvageable tissue > 6 h)	NIHSS ≥ 6 (median 10); 71 y	90-d mRS shift → mRS 0–2 (prespecified)	58.6% vs. 46.6% (aRR 1.24, 1.07–1.44)	ICH 11.4% vs. 6.0%; sICH 4.7% vs. 2.2%
DISTALS (2026)	RCT, device-specific; 118 randomized	Distal/medium vessel occlusion; ≤24 h; no IVT	Unrestricted	CTP reperfusion without sICH	86.3% vs. 27.7% (*P* < 0.001)	No sICH in device arm
MEMENTO registry (2025)ᵃ	Observational, 15 MENA centres; 164	M2, M3, A2-3, P2-3; 80% ≤6 h	Median NIHSS 10; 72 y	mRS 0–2 at 90 d (single arm)	54% independent; TICI ≥2b 69%; modelled ICER $26,875–71,333/QALY	sICH 3.0%; mortality 11%

ᵃ Single-arm registry without concurrent medical-management controls; comparisons with trial control arms are indirect and hypothesis-generating only

*acOR* adjusted common odds ratio, *aRR* adjusted risk ratio, *CTP* CT perfusion, *EVT* endovascular thrombectomy, *ICER* incremental cost-effectiveness ratio, *ICH* intracranial hemorrhage, *IVT* intravenous thrombolysis, *MENA* Middle East and North Africa, *mRS* modified Rankin Scale, *NIHSS* National Institutes of Health Stroke Scale, *QALY* quality-adjusted life year, *QoL* quality of life, *RCT* randomized controlled trial, *sICH* symptomatic intracranial hemorrhage

### The 2025 Trials: Three Neutral Answers

DISTAL randomized 543 patients (median age 77, median NIHSS 6) at 55 hospitals within 6 h, or 6–24 h with salvageable tissue; the 90-day modified Rankin Scale (mRS) distribution did not differ, EVT did not increase symptomatic intracranial hemorrhage (sICH) or death, and the natural history of MeVO stroke proved worse than expected [8]. Twelve-month data confirmed neutrality (adjusted common OR 0.81, 95% CI 0.59–1.12; survival hazard ratio 1.46, 95% CI 0.93–2.30) without quality-of-life gain [14].

ESCAPE-MeVO randomized 530 patients within 12 h; mRS 0–1 at 90 days occurred in 41.6% with EVT versus 43.1% with usual care (adjusted risk ratio 0.95), with higher sICH and mortality in the EVT arm [9].

DISCOUNT was halted at its first interim analysis after 163 of 488 planned patients: 90-day mRS 0–2 in 60% versus 77% (OR 0.42, 95% CI 0.2–0.88), with more serious adverse events (39% vs. 31%), any intracranial hemorrhage (44% vs. 29%) and sICH (12% vs. 6%) [10, 15]. The final report confirmed no improvement in three-month outcome and more hemorrhagic complications [10].

Pooled across the three trials (1246 patients), odds ratios were 0.92 for excellent, 0.87 for good and 0.84 for favorable outcome — no difference — with heterogeneity driven almost entirely by DISCOUNT [13]; a second meta-analysis found significantly more serious adverse events with EVT (RR 1.3, 95% CI 1.1–1.5) [16]. The honest summary of 2025 was: no benefit, probably some harm, considerable residual uncertainty [17].

### Why Were the First Trials Neutral? Five Explanations

#### The Deficits Were Mild and The Patients Old

DISTAL’s median NIHSS was 6 [8], and median ages of 74–77 years exceeded the pivotal LVO trials [18]. When ~ 43% of controls already reach mRS 0–1, there is little room for incremental benefit and ample room for procedural harm. Multicentre data had already suggested that MeVO thrombectomy outcomes differ meaningfully above and below an NIHSS threshold of 6 [19].

#### Reperfusion was Incomplete

In ESCAPE-MeVO only 52.2% of EVT patients achieved meTICI 2c–3; infarct volumes were smaller in those patients than in controls, yet 90-day mRS did not differ (adjusted common OR 1.17, 95% CI 0.79–1.75) [20]. Even technically successful MeVO EVT did not reliably translate into function.

#### The Tools Were Not Built for the Target

None of the devices used in the three trials was designed for medium vessels — an echo of the IMS III era — and distal tortuosity, mobility and fragility raise perforation and hemorrhage risk [15, 21].

#### Time, Access and Workflow

The ESCAPE-MeVO investigators noted difficulty accessing distal occlusions, which delayed reperfusion; late, difficult reperfusion may simply be futile reperfusion [9].

#### Endpoints and Equipoise

A DISTAL substudy found EVT increased the probability of tissue preservation, offering a different lens on treatment success [22]: if tissue is saved but the mRS does not move, either the tissue is functionally silent or the endpoint is insensitive. Meanwhile registry data continued to look favorable — one multicentre series reported better 90-day mRS with EVT (adjusted common OR 1.38), particularly in more severe strokes [23] — raising the possibility that patients most likely to benefit were treated outside the trials [7]. In the ESCAPE-MeVO imaging analysis, collateral status was the imaging variable associated with outcome (adjusted common OR 0.82) [24].

## Real-World Validation: What the MEMENTO Registry Adds

MEMENTO retrospectively analyzed consecutive DMVO thrombectomies (M2, M3, A2-3, P2-3) at 15 comprehensive stroke centres between 2022 and 2024, spanning high-income (25%), upper-middle-income (50%) and lower-middle-income (25%) settings. Median age was 72 years, median NIHSS 10, and 37% received IVT. Successful recanalization (TICI ≥2b) was achieved in 69%, 90-day functional independence in 54%, first-pass effect in 32%, sICH in 3.0% and 90-day mortality in 11% [2].

The concordance with the randomized trials is the central finding. Outcomes and — importantly — the 3% sICH rate closely mirrored the trial arms despite markedly different health systems, operator volumes and imaging availability [2]. Our interpretation, stated in the primary report, is deliberately deflationary: the numerical difference between our 54% independence rate and the ~ 45% pooled trial control estimate does not establish benefit, because it is an indirect comparison vulnerable to selection bias [2]. What the registry does establish is external validity — the neutral trial results are unlikely to be an artifact of trial conduct or of high-resource settings.

Three further observations have practical value.

### Prognostic Factors are not Treatment-Effect Modifiers

Independent predictors of poor 90-day outcome were age > 75 (aOR 2.34), NIHSS > 15 (aOR 3.12), unsuccessful recanalization (aOR 4.89) and baseline secondary subtype (aOR 2.08); neither technique nor imaging modality predicted outcome [2]. We stress the distinction we drew in the original paper: without a concurrent control arm, these identify who does badly after thrombectomy, not who fails to benefit from it. Age > 75 is a prognostic marker, not a contraindication.

### Recanalization Is Necessary but not Sufficient

Failed recanalization carried the largest effect size in our model [2], and first-pass effect has been repeatedly linked to better outcomes and less infarct growth in DMVO [25]. Yet ESCAPE-MeVO showed that achieving near-complete reperfusion did not secure functional benefit [20]. The two findings are compatible and jointly instructive: reperfusion is a prerequisite for benefit but does not guarantee it, so the goal must be *fast*,* first-pass*,* atraumatic* reperfusion in patients with enough at stake to justify the attempt.

### Technique May Matter more than Assumed

Stent retriever achieved the highest recanalization (85%), followed by combination (74%) and contact aspiration (45%; p = 0.046), with a parallel gradient in functional independence (68%, 58%, 34%) that did not reach significance; combination technique was fastest (35 vs. 40–42 min) [2]. We present this cautiously — allocation was at operator discretion, aspiration-first was likely chosen for the most distal and tortuous targets, and confounding by indication is unavoidable. The signal nonetheless runs against series favoring aspiration or combination approaches [26, 27], aligns with location-specific device experience [28], and deserves a dedicated randomized technique comparison rather than another observational hierarchy.

We also adjudicated TICI centrally using a locally developed, open-source AI engine with human override, validated against blinded manual scoring in 24% of cases (κ = 0.84) [2]. Our practical lesson: automated, auditable reperfusion grading substantially shortens adjudication time and constrains the optimistic drift that makes single-arm technical outcomes hard to trust. Future MeVO trials should consider it — the modest hemorrhagic phenotypes typical of distal intervention, including petechial hemorrhage, likewise warrant standardized adjudication rather than operator report [29].

### The Economics of a Marginal Indication

No randomized MeVO trial has reported cost. In MEMENTO we modelled cost-effectiveness using published control-arm outcomes as external benchmarks, yielding incremental cost-effectiveness ratios of $26,875/QALY (lower-middle income), $45,200/QALY (upper-middle income) and $71,333/QALY (high income) — 7.0-, 3.5- and 2.5-fold exceedances of WHO-recommended GDP-per-capita thresholds, with 0% probability of cost-effectiveness on probabilistic sensitivity analysis and a budget impact of $11.2–19.4 million per million population, equal to 2.8–7.1% of total health expenditure [2]. For context, LVO thrombectomy sits comfortably at roughly $10,000–40,000/QALY [2].

We were explicit in the primary paper that these figures are illustrative rather than definitive: they rest on an assumed 9% absolute benefit that randomized trials do not support, so the true ICER is plausibly worse — potentially undefined if the incremental benefit is zero [2]. Two internal caveats belong in any citation of this work: the estimates depend on historical controls, and the number-needed-to-treat derivation is sensitive to which benefit assumption is used.

The policy inference survives the uncertainty. In a health system without a functioning 24/7 LVO service, an organized stroke unit or a secondary-prevention programme, expanding into medium vessels is close to indefensible on opportunity-cost grounds alone. This is not a MENA-specific argument; it applies to any system where the marginal stroke dollar has a higher-yield alternative. It is, however, an argument that could only be made from data collected across income strata.

### 2026: ORIENTAL-MeVO and the Return of a Treatment Effect

ORIENTAL-MeVO randomized 563 patients at 48 Chinese centres within 24 h of a moderate-to-severe MeVO stroke (NIHSS ≥ 6; salvageable tissue required beyond 6 h) [11, 30]. Median age was 71, median NIHSS 10, and 36.6% received IVT [11]. Because the proportional odds assumption was violated, 90-day functional independence became the primary endpoint as prespecified: 58.6% versus 46.6% (adjusted rate ratio 1.24, 95% CI 1.07–1.44; *P* = 0.004) [11, 31]. Secondary endpoints concurred — mRS 0–1 in 48.9% versus 33.2%, and vessel patency at 24–72 h in 82.1% versus 46.2% — while radiological intracranial hemorrhage was more frequent with EVT (11.4% vs. 6.0%) and mortality (11.1% vs. 10.2%) and sICH (4.7% vs. 2.2%) did not differ statistically [31]. For every 100 patients treated, 54 had a less disabling outcome and 12 additional patients became independent [31]. Nogueira’s framing of the trade-off was memorable: “We do pay a price for thrombectomy” — a price he judged worth paying given low absolute hemorrhage numbers [32].

Why did ORIENTAL-MeVO succeed? Its distinguishing feature was severity: DISTAL and ESCAPE-MeVO admitted patients with low NIHSS scores, cited as a principal reason for their neutrality [31, 33]. The accompanying editorial titled the message plainly — narrowing the target population [34]. Notably, this threshold converges with what our registry showed independently: NIHSS > 15 predicted poor outcome, but the bulk of our cohort sat at a median NIHSS of 10, squarely within ORIENTAL-MeVO’s enrolment band, and achieved 54% independence [2].

Two caveats temper enthusiasm. Control-arm independence was 46.6% in ORIENTAL-MeVO versus 77% in DISCOUNT [10, 11] — different populations, different systems, and confirmation outside China is required. And imaging selection is not a panacea: a post hoc analysis found baseline CT perfusion was not associated with better functional outcomes (adjusted OR 1.07, 95% CI 0.79–1.45) though it was associated with lower mortality (7.2% vs. 13.9%; adjusted RR 0.57, 95% CI 0.35–0.94) [35]. Our registry pointed the same way: perfusion imaging was used in 60% of patients and was neither predictive of outcome nor associated with better results than ASPECTS-based selection [2].

### Device–Vessel Matching: The DISTALS Trial

If technical failure explains part of the 2025 signal, the device is a modifiable variable. DISTALS randomized 118 patients within 24 h to thrombectomy with a low-profile stentretriever purpose-built for distal vessels — a device with a decade of dedicated DMVO experience behind it [36] — or medical management alone, deliberately targeting patients who had not received IVT [12]. Successful reperfusion without sICH occurred in 86.3% versus 27.7% (*P* < 0.001), with zero sICH in the device arm; at 90 days more than twice as many device-treated patients reported a full return to normal quality of life [12, 37].

We read this cautiously: the trial is small, industry-sponsored, and its primary endpoint is a tissue-level surrogate rather than disability [33]. Yet it establishes the premise the earlier trials lacked — with matched hardware, MeVO reperfusion is achievable reliably and apparently safely. Device–vessel matching combined with patient selection is the plausible route to clinical benefit [38], and it directly addresses the 45% aspiration recanalization rate we observed in the field [2].

### Medical Management is not a Placebo Arm

Any decision to intervene must be benchmarked against improving medical therapy. Roughly two-thirds of DISTAL patients and 37% of ours received IVT [2, 8], and the natural history of MeVO with and without alteplase is well characterized [39]. Intensifying lysis in mild syndromes has not worked: tenecteplase did not improve outcomes in minor stroke with proven occlusion in TEMPO-2 [40]. Adjunctive intra-arterial lysis after reperfusion improved LVO outcomes in CHOICE [41] and is an attractive MeVO strategy, with a dedicated trial of intra-arterial tenecteplase now registered [42]. In our experience the most under-used medical decision is the simplest: withholding intervention in a patient improving rapidly after IVT.

### How We Decide Today: A Pragmatic Framework

**Fig. 1:**
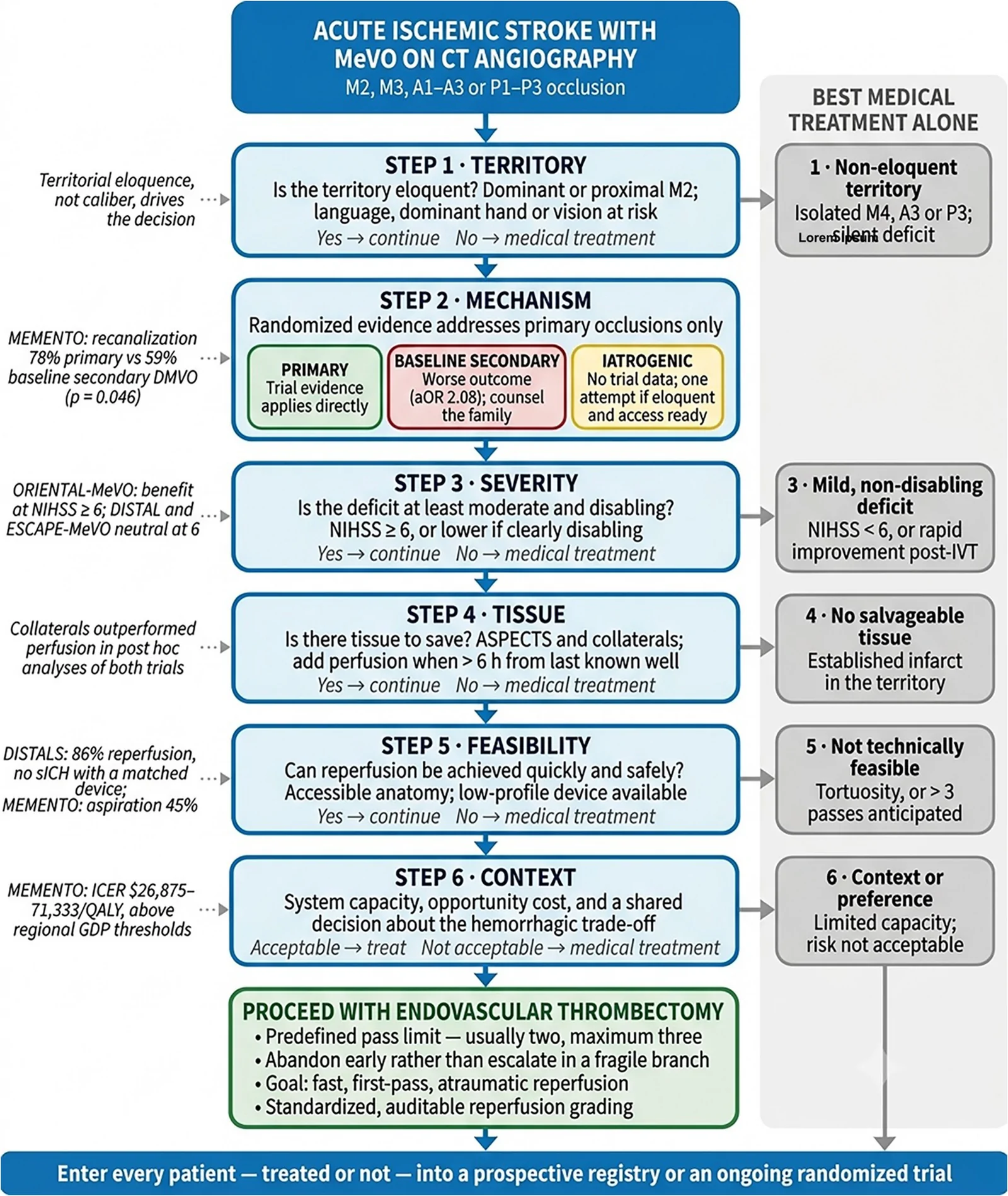
Proposed decision framework for acute medium vessel occlusion (MeVO) stroke in the post-trial era. Step 1: confirm MeVO on CT angiography and classify by territorial eloquence (dominant/proximal M2 and eloquent cortex versus non-eloquent M4/A3/P3). Step 2: determine occlusion mechanism — primary, baseline secondary (proximal clot fragmentation) or iatrogenic secondary (peri-procedural embolus) — since randomized evidence addresses primary occlusions only. Step 3: grade clinical severity (NIHSS ≥ 6 or clearly disabling deficit)

MeVO is not a treatment indication; it is an anatomical description. Six questions decide our cases (Fig. 1; Table 2).

**Table 2:** Factors shifting the balance for or against thrombectomy in medium vessel occlusion stroke

Domain	Favors thrombectomy	Favors medical management alone
Clinical severity	NIHSS ≥ 6, or lower with clearly disabling deficit	NIHSS < 6 and non-disabling deficit; rapid improvement after IVT
Territory / site	Dominant or proximal M2; eloquent cortex (language, dominant hand, vision)	Isolated M4, A3, P3; clinically silent territory
Occlusion mechanism	Primary occlusion (highest recanalization, best outcomes); iatrogenic embolus in eloquent branch with access already established	Baseline secondary occlusion from proximal clot fragmentation (independent predictor of poor outcome)
Imaging	Salvageable tissue; poor collaterals with viable penumbra	Established infarct in symptomatic territory; excellent collaterals with improving deficit
Time and workflow	Early presentation; short anticipated access time; in-house neurointervention	Late presentation with long anticipated procedure; transfer delay
Technical feasibility	Accessible anatomy; purpose-built low-profile device or stentretriever available	Extreme tortuosity; aspiration-only platform in a very distal branch; >2–3 passes anticipated
Patient factors	Independent premorbid status; patient/family accepts hemorrhagic risk	Frailty, high hemorrhage risk, conservative goals of care
System / resource context	Mature LVO service, stroke unit and prevention programme already in place	Incomplete LVO capacity or stroke-unit provision, where opportunity cost of MeVO expansion is high

*IVT* intravenous thrombolysis, *LVO* large vessel occlusion, *MeVO* medium vessel occlusion, *NIHSS* National Institutes of Health Stroke Scale

#### Is the Deficit at Least Moderate and Disabling?

We use an informal threshold of NIHSS ≥ 6 after ORIENTAL-MeVO [11], lower when the deficit is clearly disabling (severe isolated aphasia, dominant-hand paresis, hemianopia in a driver). For NIHSS 3–5 non-disabling syndromes we default to medical therapy: DISTAL and ESCAPE-MeVO indicate the expected gain is small while the procedural risk is not [8, 9].

#### Is the Territory eloquent, and the Occlusion Proximal enough to Matter?

 Dominant or proximal M2 occlusions behave like small LVOs; isolated M4, A3 or P3 occlusions do not [1].

#### What is the Mechanism Primary or Secondary?

For primary occlusions the randomized evidence applies. For iatrogenic distal embolization discovered mid-procedure, no trial applies; we set a low threshold for one careful attempt when the branch is eloquent and access is already established, and a hard stop otherwise. For baseline secondary occlusions we counsel families that outcomes are systematically worse [2].

#### Is there Tissue to Save? 

Perfusion does not identify responders automatically [2, 35], and collateral status may be the more informative variable [24]; we weight time from onset heavily given the ESCAPE-MeVO access-delay signal [9].

#### Can we Get there Quickly and Stop in Time?

We commit to a distal-access plan before groin puncture, prefer purpose-designed low-profile devices where available [12, 38], and set an explicit pass limit (usually two). The single most useful discipline we have adopted since 2025 is abandoning the procedure early rather than escalating in a fragile M3 branch [15]. Where only aspiration platforms are available, our data argue for realism about achievable recanalization [2].

#### What does this Cost the Next Patient?

In settings where LVO capacity, stroke-unit beds or prevention programmes are incomplete, the answer to “should we treat this MeVO?” is partly institutional rather than clinical [2]. We regard resource context as a legitimate input, and we log every MeVO — treated or not — in a prospective registry.

### Guidelines and Real-World Practice

The 2026 AHA/ASA guideline emphasizes rapid reperfusion, streamlined imaging and avoidance of interventions shown to provide no benefit or cause harm [43]. While strengthening recommendations elsewhere, the writing group flagged medium and distal vessels as a domain where evidence is thin and current technology may underperform [44]; clinicians should read the MeVO recommendations directly, as they distinguish occlusion subgroups. Practice remains heterogeneous [7], and anesthesia strategy, adjunctive antithrombotics and rescue after failed IVT are all unresolved.

### Research Agenda

Four priorities seem non-negotiable. Endpoints: MeVO trials need measures more sensitive than dichotomized mRS, including utility-weighted scales and tissue-level outcomes [12, 22]. Pooling and replication: an individual-patient-data meta-analysis across all five trials is the fastest route to identifying responders, and ORIENTAL-MeVO requires replication outside China [1, 11]. Registry-embedded randomization and economic endpoints: the deficiency of our own study — no concurrent control — is remediable through parallel medically managed cohorts or randomization nested within registries, and equipoise plainly exists for low-NIHSS, very distal and resource-limited scenarios [2]. Selection science and technology: machine-learning prognostication [45], purpose-built devices [12, 46], intra-arterial lysis [42] and perfusion-guided late-window randomization [47] are all in progress. A randomized comparison of stent retriever versus aspiration versus combination in medium vessels remains, in our view, the most easily executed and most immediately useful trial not yet done [2].

## Conclusions

The answer to “MeVO: to treat or not to treat?” has changed twice in eighteen months, and the change is instructive rather than embarrassing. DISTAL, ESCAPE-MeVO and DISCOUNT showed that indiscriminate thrombectomy for medium and distal occlusions — elderly patients, mild deficits, devices built for larger arteries — does not reduce disability at 90 days or at one year and increases hemorrhage and serious adverse events [8–10, 14, 16]. Our MEMENTO registry showed that this conclusion travels: across 15 centres and three income strata, real-world outcomes closely mirrored the trial arms, and the economic case for routine implementation was unfavorable at every income level [2]. ORIENTAL-MeVO then showed, with equal rigor, that restricting treatment to moderate-to-severe deficits with salvageable tissue yields a clinically meaningful gain in independence at an acceptable hemorrhagic cost [11, 31]. DISTALS supplied the missing technical premise: reperfusion can be achieved in most patients, without symptomatic hemorrhage, when the device matches the vessel [12, 37].

Taken together, the evidence supports neither reflexive treatment nor abandonment, but a narrower and better-executed indication: a disabling deficit, an eloquent territory, salvageable tissue, a primary or accessible clot, a purpose-built device, a hard pass limit, and honest attention to what the intervention costs the patients who never reach the angiography suite. The era of expanding thrombectomy indications by anatomy alone has probably ended; the era of expanding them by mechanism, physiology and technology is beginning. Until pooled individual-patient analyses and confirmatory trials arrive, the most useful thing to do with the next MeVO patient is neither to treat nor to withhold on instinct, but to select carefully — and wherever possible, to randomize.

## Key References

- Jing X, Nogueira RG, Hu W, et al. Endovascular treatment of medium-vessel-occlusion strokes. N Engl J Med. 2026. https://doi.org/10.1056/NEJMoa2514120 .
  - *○ The first randomized trial to demonstrate benefit of thrombectomy in MeVO stroke*,* achieved by restricting enrolment to moderate-to-severe deficits (NIHSS ≥ 6)*,* with 90-day independence of 58.6% versus 46.6%. It reframes the MeVO question from “whether” to “in whom.”*
- Clarençon F, Bala F, Baptiste A, et al.; DISCOUNT Investigators. Mechanical thrombectomy in ischemic stroke with a medium or distal arterial occlusion: the DISCOUNT randomized clinical trial. JAMA. 2026. https://doi.org/10.1001/jama.2026.8977 .
  - *○ A trial stopped early amid futility and safety concerns*,* showing no improvement in three-month outcome and more hemorrhagic complications with thrombectomy. It is the strongest cautionary counterweight to enthusiastic MeVO intervention.*
- Mansour OY, Ozdemir AO, Gurkas E, et al. Real-world outcomes of thrombectomy for distal medium vessel occlusions in the Middle East and North Africa region: a multicenter registry analysis. Neuroradiology. 2025;67(11):3363–76.
  - *○ A 15-centre*,* 164-patient registry showing that real-world DMVO thrombectomy outcomes closely mirror the neutral randomized trials across three income strata*,* while modelled cost-effectiveness ratios exceed regional thresholds by 2.5- to 7-fold. It supplies the external-validity and health-economic dimensions absent from the trials.*

## Data Availability

No datasets were generated or analysed during the current study.
